# Parent–child interaction during a home STEM activity and children’s handwashing behaviors

**DOI:** 10.3389/fpsyg.2022.992710

**Published:** 2022-11-17

**Authors:** David M. Sobel, Laura W. Stricker

**Affiliations:** ^1^CLPS Department, Brown University, Providence, RI, United States; ^2^CLPS Department, Providence Children’s Museum, Brown University, Providence, RI, United States

**Keywords:** parent–child interaction, handwashing, prevention of disease transmission, STEM engagement, goal setting

## Abstract

We examined correlations between a home-based STEM activity illustrating the importance of soap use during handwashing and children’s (4-to 7-year-olds, *N* = 81, 42 girls, 39 boys) use of soap when washing their hands. Parents and children either participated in or watched the activity. Children reflected on the activity immediately afterward and a week later. Parent–child interaction during participation related to the frequency of unprompted soap use during handwashing, controlling for performance on other, related cognitive measures. Children whose parents were more goal-directed, and set goals for the interaction, were less likely to use soap spontaneously when handwashing in the subsequent week. The amount of causal knowledge children generated when they reflected on the experience immediately afterward also influenced whether children used soap when washing their hands. Reducing the autonomy children believe they have during a STEM-based activity potentially leads them to not engage in a behavior related to the activity on their own. Overall, these data suggest that parent–child interaction during STEM activities can influence the ways children encode and engage with those activities in their everyday lives. Given that the ways children wash their hands might mitigate the spread of disease, interventions that focus on providing children with the belief that STEM activities are for them might be broadly beneficial to society.

## Introduction

Collaborative, playful interaction is an essential part of young children’s learning experience (e.g., Weisberg et al., 2014). Hands-on informal learning environments, like museums, offer rich ecosystems for studying these kinds of interactions, allowing researchers to capture children’s play, STEM exploration, and parent–child engagement in a naturalistic way (e.g., Allen, 2004; van Schijndel et al., 2010; Callanan, 2012; Sobel and Jipson, 2016; Falk and Dierking, 2018). But informal learning happens in many contexts (e.g., Ridge et al., 2015; Hassinger-Das et al., 2018, 2020; Gaudreau et al., 2021; Morris et al., 2021), and most critically in the home. Our goal is to examine the translation of parent–child interaction practices in hands-on museum settings to similar hands-on STEM-based activities in the home to consider whether there are corresponding learning outcomes from those interactions.

Parent–child interaction has been studied in the home in many ways, particularly through observational means (Parker et al., 1999; Aspland and Gardner, 2003; Raikes et al., 2006; Deak et al., 2014). Parents support children’s learning through various facets of their cognition and language (e.g., Tamis-LeMonda et al., 2001, 2004; Song et al., 2014). Our goal is to build upon this observational work and use our findings to contribute to museums’ practices for engaging families outside their physical space. To do this, we presented families with a STEM-based activity, developed in conjunction with museum educators, to be done in the home. We then examined parent–child interaction during this activity, following other investigations of parent–child interaction in museum settings, and then measured children’s subsequent reflection about the activity and their engagement with real-world behaviors as it relates to the activity.

Because of its relevance to the COVID-19 pandemic (and to mitigating the spread of disease in general), we have chosen children’s handwashing as the behavior of interest, and particularly, their use of soap during handwashing to prevent germ transmission. For older children (6^th^ graders), studies have shown that the way in which parents wash their hands, and the nature of the bond between parents and children, relates to children’s own handwashing behavior (Song et al., 2013). Children’s beliefs about germs, how germs spread, and how to mitigate this spread is not only relevant to promoting good hygiene, but also preventing the spread of infectious diseases (Au et al., 1999, 2008). Observational studies of children’s handwashing have shown that 3-to 6-year-olds wash their hands before eating, after outdoor play and after bathroom use only 15–48% of the time in daycare settings (van Beeck et al., 2016, see also Toyama, 2016a). Educating young children on the importance of soap use during handwashing reduces the physical number of bacteria on their hands (Kim et al., 2012; Utario et al., 2018). Educating young children on germs and handwashing also increases their understanding of the relation between germs and disease prevention (e.g., Toyama, 2016b; Crosby et al., 2019; Jess and Dozier, 2020; Younie et al., 2020 for a review). This literature, however, does not highlight any case in which the interaction between parents (or teachers) and children influenced children’s subsequent handwashing behavior.

Our question was whether presenting families with an activity that highlights what soap does during handwashing affects children’s handwashing behaviors, compared with watching that activity on video. Subsequently, we also considered whether the ways in which parents and children interact during this activity relates to children’s use of soap during subsequent handwashing. To this end, we first highlight studies on digital learning and its similarities and differences to hands-on demonstrative learning. We then consider how parent–child interaction during hands-on activities might affect children’s learning and engagement.

## STEM learning from digital media

Digital educational resources, which include online games, websites, apps, and videos, are thought to serve as an effective way to engage children in STEM learning—both in and out of the classroom. In the home, parents often use these kinds of media as a supplemental tool to reinforce kid-friendly math and science concepts; the use of internet videos, in particular, is a way to visually aid children’s science inquiry and encourage scientific curiosity (Hightower et al., 2019). In elementary schools, teachers see digital-based resources as a way to reenergize the classroom curriculum; providing real-world relevance and a different, more unconventional way of reaching students (Hanson and Carlson, 2005). From videos, young learners are able to experience concrete, visual examples of the content being explored (Nugroho and Muhtadi, 2020). Video learning also positively impacts children’s performance, participation, and interest in scientific topics (e.g., Giannakos et al., 2015; Palaigeorgiou and Papadopoulou, 2019; Chen et al., 2021). As such, it is possible that watching videos of the same STEM demonstrations affords children the same learning opportunities as participating in those demonstrations themselves. In our study, we contrasted dyads who participated in the STEM demonstration with those who watched that demonstration for the purpose of comparing these groups.

## Hands-on STEM learning and parent–child interaction

For decades, however, hands-on museums and science centers have focused on providing the public with exploratory and participatory STEM learning experiences. The rationale for this pedagogy, led by Oppenheimer (1968), is that verbal explanation of science concepts alone is not enough to initiate understanding. Hands-on STEM activities engage visitors with real objects and phenomena and encourage active participation through autonomy, initiative and choice (Caulton, 2006). For children, opportunities to explore museum exhibits through hands-on manipulation increases their time spent engaging with STEM content (Crowley et al., 2001a; Knutson et al., 2016; Willard et al., 2019). For example, families’ interaction with a natural history museum’s diorama increased significantly after the implementation of a range of hands-on interventions. One of the most successful interventions was “Objects and Tools” which featured real-life specimens and investigative tools (deer antlers, jaw bones, measuring tools, etc.) that families could explore freely in tandem with the diorama (Knutson et al., 2016). Additionally, hands-on objects can increase joint talk between parents and children and encourage children’s experience recall after their visit (Jant et al., 2014). These benefits of hands-on exploration are made even stronger when the experience is a collaborative one, with scaffolded support from an adult (e.g., Crowley et al., 2001a; Van Schijndel et al., 2010; Jant et al., 2014; Legare et al., 2017; Willard et al., 2019).

In particular, the ways in which parent–child interaction scaffolds children’s STEM learning and engagement has been studied in three ways. First, how children and parents talk to each other during the activity affords meaning construction and the transmission of causal knowledge (e.g., Callanan and Jipson, 2001; Leinhardt and Knutson, 2004; Eberbach and Crowley, 2017). For example, elaborative talk about science in informal settings, such as parents generating explanations and asking open-ended questions, relates to children’s engagement with exhibits and to their ability to remember more about their experience (e.g., Benjamin et al., 2010; Jant et al., 2014). The explanations parents generate provide a structure for the activities that children engage in, which may help children better understand the information inherent in the exhibit (e.g., Crowley et al., 2001b; Tare et al., 2011; Callanan et al., 2020; Chandler-Campbell et al., 2020, although see Joy et al., 2021, for a different perspective).

For example, in a children’s museum tinkering space, the more parents generated STEM-based talk while engaging in a tinkering activity with children, the more likely children were to talk about STEM-related content when asked to reflect on the activity (Acosta et al., 2021). Similarly, encouraging parents to promote spatial talk with their preschoolers led to preschoolers generating more spatial language during their play, and the extent to which children generated such language related to their spatial problem solving on their own (Polinsky et al., 2017). Generally construed, the more science talk parents generate when exploring an exhibit (in this case, discovering the identity of a novel object), the more engaged children were by the activity (Valle and Callanan, 2006; Haden, 2010; Callanan et al., 2017), and the more personal connections parents make for children during their conversations at exhibits, the longer children spend exploring the exhibit (e.g., Crowley et al., 2001a; Pattison et al., 2018).

Second, how parents set goals or allow children to set goals during play relates to children’s engagement with the interaction they have with their parents. For example, Sobel et al. (2021) showed that parents who were more directive in setting goals during free play at a circuit exhibit had children (specifically 4-to 7-year-olds) who participated in fewer circuit building challenges, controlling for age and how well children performed at building circuits. Similarly, Leonard et al. (2021) similarly found that when adults “take over” their interaction with children during a challenging task – i.e., when adults engage in the task for the child – those children were rated as persisting less on a measure of global persistence. These researchers also found that children engaged with stimuli for less time on their own when an adult experimenter took over the interaction than when the adult engaged in other activities (see also Medina and Sobel, 2020, for similar findings when children engage in a learning activity with a caregiver).

An interpretation of these studies is that when parents reduce children’s autonomy during interaction, children become less engaged with the activity and are less likely to internalize and encode their participation. Such a possibility has support in the adult social psychological literature, as well as in parent–child interactions among older children. Deci and Ryan (2000), for instance, suggest that the extent to which adults feel they have autonomy in their actions – that they can “self-organize experience …and to have activity be concordant with one’s integrated sense of self” (p. 231) – the more they engage in healthy development and experience well-being. Applying this hypothesis to children, Grolnick & Ryan, 1987 found that when adults placed fifth-graders in a directed learning environment that controlled what children were allowed to do (by indicating that their participation was a test and that they should work hard), their motivation for learning was reduced, compared with a case in which less controlling and evaluative language was used. In formal academic settings, the extent to which parents supported their 3rd to 6th graders’ autonomy positively correlated with children’s self-regulatory behaviors and academic achievement (Grolnick & Ryan, 1989). While we build on more recent studies of younger children’s interaction with parents during informal learning activities, the negative influence of “taking over” behaviors or of parents’ goal directedness has its basis in the rubric of the social psychology of self-determination.^1^

Third, how children reflect on informal learning experiences with their parents after the fact indicates what they understand about the experience (e.g., Haden, 2010). For example, if causal information is presented to children during their interaction with parents in a museum setting, children talk more about that causal knowledge when they reflect on the experience even 2 weeks later (Marcus et al., 2017). Reflection also promotes consolidation, which can be applied to subsequent activities. Marcus et al. (2021) showed that having parents and children reflect on their play at a museum exhibit together related to children’s understanding of the engineering knowledge inherent in the exhibit when children were tested in the home a week later. This suggests that parent–child interaction and the ways in which children reflect on the experience in the museum not only transfers to the home environment, but also that reflection on such experiences relates to how children are motivated to engage in tasks and problem-solve more generally. The more causal knowledge children might have, the more likely they might be to internalize and apply their experiences during parent–child interaction to other facets of their lives.

## The present study

In the present study, we asked parents and children to engage in a structured activity. The activity we used centered around demonstrating the effect that soap has on particles in water. Of importance is whether children encode the difference between using and not using soap during their experience, as well as whether parents set goals for their children’s participation in the activity, thus increasing or decreasing children’s perceived autonomy. Our specific hypothesis was that parents who engaged in more goal-setting behaviors would have children who showed reduced engagement with what could be learned from the measure. To provide a baseline, we also had a separate group of parents and children watch the activity on a video, so that children were exposed to the content of the activity, but without the possibility of controlling their behavior during participation.

Conversations between parents and children were recorded during and immediately after their participation in the activity or their watching of the demonstration video. Children were also asked to reflect on their experience with the demonstration in the same session and approximately 1 week later in a separate session. Additionally, in both sessions, children were given a set of measures to control for their general cognition and to assess their understanding of disease transmission. This ensured that any difference we potentially observed between conditions related to the conditions and not children’s existing causal knowledge or cognitive capacities. During the time between the two sessions, parents were sent a daily Google Form, in which they were asked to reflect on one observation of their children’s handwashing behaviors that day – particularly whether they washed their hands before eating or after bathroom use and whether they used soap. Summary statistics from these reports will constitute our dependent measure, and we will consider whether facets of parent–child interaction, children’s reflection, and their knowledge of disease transmission influence this handwashing behavior.

For the at-home STEM activity, we chose a demonstration in which grains of black pepper are placed in a bowl of water, and displaced when soap (particularly soap on a finger) is put into the bowl. Children either observed a video of the demonstration or physically participated in it, and through this experience, were able to see what happened when they or another person dipped their finger into the bowl without, and then with the soap. Without soap, the pepper sticks to the person’s finger. With soap, the pepper moves away from the person’s finger, as if repelled. Of course, this is not the actual causal mechanism – the soap does not repel the pepper; rather, the soap breaks the surface tension of the water because one end of the soap molecule is hydrophobic. However, the goal of this demonstration is not to teach children about surface tension.^2^ Rather, the goal is to present children with a scenario in which using soap affects how they might visualize and represent germs sticking to their body, a fact that even the youngest children of this age can denote through symbolic representation (e.g., DeLoache, 1987). Critically, the movement of the pepper is fast and surprising, creating an engaging result, which is easily perceptually accessible.

As such, there are three research questions we wish to address. First, does the way parents and children interact during their participation in the activity relate to children’s subsequent handwashing behaviors? We look at this in two ways: by considering whether there is a difference between dyads who actually participated in the activity and those who watched a video of the activity and by examining whether parental goal setting during the activity mediated handwashing behavior in the former group. Of interest was whether any hands-on participation would facilitate children’s handwashing behavior or if they specifically needed the activity to be non-parent-directed. This question also motivated an important facet of our investigation, which was that at no point during the demonstration or participation did we tell parents or children that the study was about children’s handwashing. We did not want to bias parents from talking about handwashing, germs, or disease prevention; rather, we wanted to see if this talk would emerge naturally. Moreover, we did not want to bias parents from enforcing handwashing or soap use after the demonstration; we similarly wanted to see whether children would engage in more handwashing or soap use on their own.

Second, does the conversation children have with their parent during the activity or their reflection on their experience with the activity relate to their handwashing behaviors? To answer this question, we focus on the causal language generated by parents and children during their participation or viewing of the activity as well as the causal language children generate during their reflections. Of particular interest here is whether the generation of causal language by parents or children, particularly about germs, handwashing, or disease transmission, during the activity related to children’s subsequent handwashing. Again, because our goal was to examine everyday parent–child interaction, we did not explicitly tell parents that the goal of the investigation was to study handwashing or soap use. This question, however, considers the extent to which parents or children’s spontaneous application of this knowledge to the situation influenced children’s subsequent behavior.

Third, are there individual differences in children’s knowledge of disease transmission, or other facets of their cognition that might moderate their handwashing behavior? Here, we consider how children respond to specific questions designed to assess their knowledge of disease transmission in general as well as broader measures of cognitive development, such as working memory and theory of mind. These measures were chosen both as measures of general cognitive development, but also because greater memory or social-cognitive capacities might moderate how one learns from parent–child interaction. The expectation was that any relations we found of interest to the research questions described above would not be due to general cognitive development, and thus unrelated to performance on the theory of mind and working memory measures.

## Materials and Methods

### Participants

The final sample included 81 children between the ages of 49 to 96 months (42 girls, 39 boys, *M_age_* = 72.45 months, SD = 13.69 months). This sample size was determined by a power analysis based on comparison between the two conditions, assuming a large effect (*f* = 0.35), and α = 0.05 and β = 0.80. Participants were recruited from a database of families who had previously participated in studies in the laboratory or children’s museum in Location Blinded for Review as well as through an advertisement on Childrenhelpingscience.org.

Children were tested over two sessions, both conducted over Zoom. In the first session, they participated in or observed the demonstration with their parent (74 with female parent, 7 with male parent), and then tested on their own. In the second session, approximately 1–2 weeks later, children were tested by themselves (after their parent established the Zoom call). Parents were invited to stay in the room while their child was tested individually, but instructed not to prompt them to respond, or respond for them. Three additional dyads were tested, but not included in the final sample. Two only participated in the first session; the third was uncooperative and did not provide a complete dataset. Participating families were compensated $20 for each session ($40 total).

We collected demographic information from participating parents *via* a self-report questionnaire which asked for parent age, household income, household language, parent education level, and family race/ethnicity information. Parents were told to provide as much information as they were comfortable sharing. All parents provided some demographic information. Seventy-two (89% of the sample) reported that their children came from monolingual English-speaking homes. Nine (11%) reported their children came from bilingual homes – always English and another language (Spanish, Portuguese, French, German, Arabic, and Tamil were represented).

Using open-ended questions, we asked parents to describe their family’s ethnicity and race. Three parents did not respond to this question. We grouped responses to the race and ethnicity questions based on the guidelines provided by NIH regarding race and ethnicity, generalizing based on parents’ open-ended responses (e.g., parents who referred to themselves as Vietnamese were categorized as Asian). Sixty-three (78% of the sample) of families that participated identified as white/Caucasian, 5 (6%) identified as more than 1 race or ethnicity, 3 (4%) identified as Asian/Asian American, 3 (4%) identified as Black/African American and 4 (5%) identified as Hispanic or Latinx. None of our families that participated identified themselves as American Indian/Alaska Native or Native Hawaiian/Pacific Islander.

Parents’ education levels fell across five categories. Twenty-five parents (31% of the sample) reported they had a Bachelor’s Degree at the time of testing. Thirty parents (37%) reported having a Master’s degree, 16 (20%) reported having a PhD (or equivalent), 4 (5%) reported having an Associate’s degree and 6 (7%) reported having some college or a High School Diploma.

Household income levels fell across six categories. Six parents (7% of the sample) did not report this information. Two parents (2%) reported a household income below $30 K. Three parents (4%) reported $31-50 K. Five parents (6%) reported $51-70 K. Eleven parents (14%) reported 71-90 K. Nineteen parents (24%) reported 91-120 K and 35 parents (43%) reported a household income of $120 K or greater.

Finally, 31 parents (38% of the sample) reported their age between 21 and 35, whilst 50 parents (62% of the sample) reported their age between 36 and 49.

In addition to providing demographics, parents were asked to complete the *Attitudes toward Science* questionnaire (Szechter and Carey, 2009), which is detailed in the section Supplementary material.

### Materials, procedure, and coding

The study procedures were approved under Brown University IRB protocol # 2005002720, *Relations Between Parent–Child Interaction During a Remote Activity and Children’s Understanding of the Importance of Hand Washing*. All families were tested in their homes *via* Zoom over two sessions. Families were randomly assigned to either the *Watch* condition (*n* = 40) or the *Participate* condition (*n* = 41), described below. We always tested one target parent and a child. Siblings and other caregivers were allowed to be present during the time that the target parent and child watched the video or participated in the demonstration, but they were not allowed to participate in the demonstration, or be present for the other portions of the session. The target parent was required to be present for the activity portion of the study. The target parent did not, however, need to be present for the remainder of the session, during which the child was interviewed. During the first session, the target parent was asked to stay nearby, because the experimenter did ask them one question at the end of the child’s reflection. The two sessions occurred between 5 and 16 days apart (*M* = 9.24 days, SD = 1.95). We will describe the procedures for the two sessions below.

#### First session

##### Demonstration: Watch vs. participate conditions

**Figure 1:**
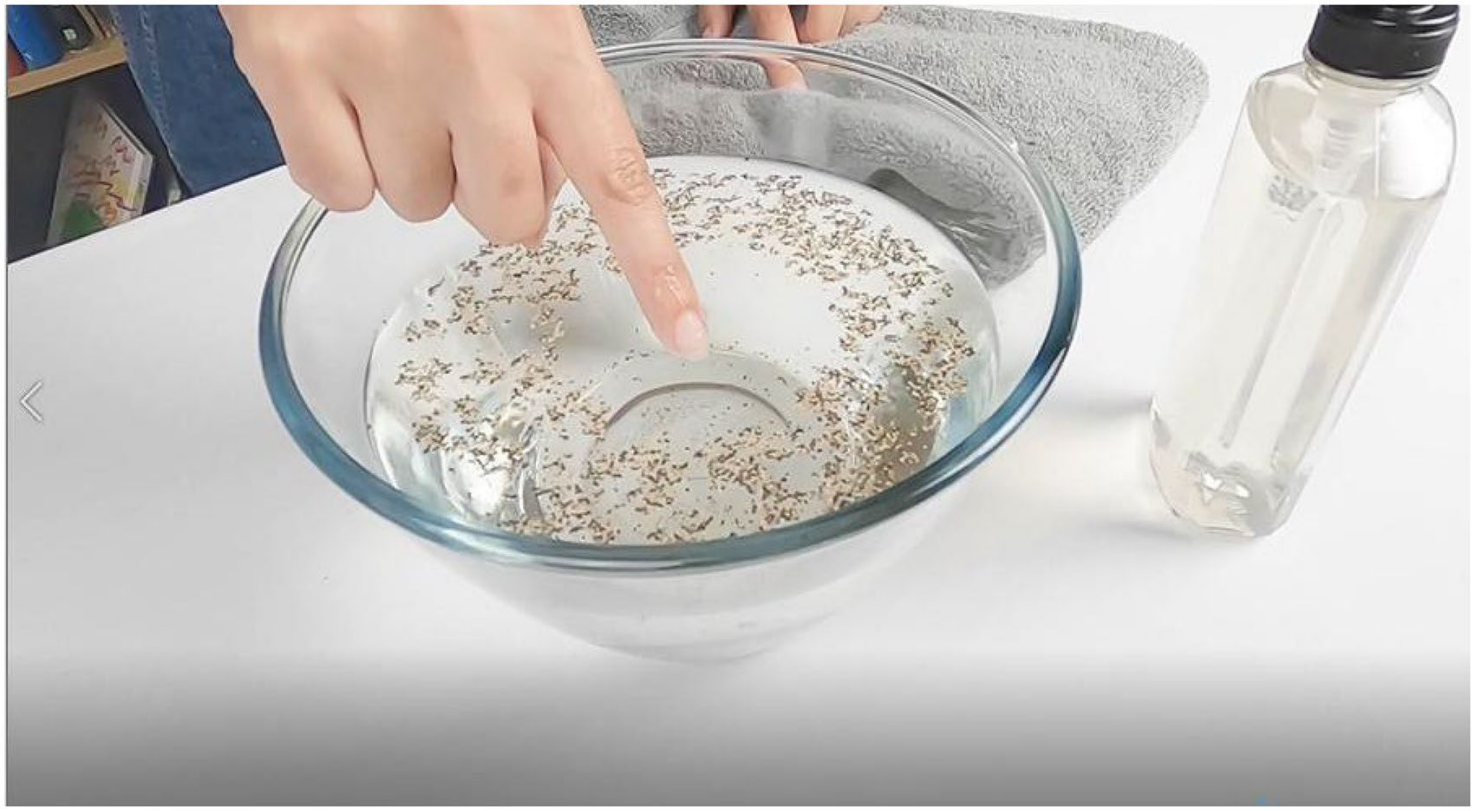
Screenshot from the video showing the reaction of the pepper to the woman’s finger with soap on it being placed in the bowl. The soap breaks the surface tension of the water, which gives the appearance of the finger repelling the pepper.

In the Watch condition, dyads watched a video of the demonstration (described below) through Zoom’s screen share function. The video depicts a woman who introduces the activity by saying “Today we are going to do an experiment. For this experiment, we will be using a bowl (a clear or light-colored bowl will work best), water, pepper, and liquid soap. We will also need a towel.” As each item is mentioned, they are brought on to the screen one at a time. The bowl is then placed on a table and the woman says, “To begin, fill the bowl with water.” On the screen, the bowl is filled with water. The woman then narrates, “Then grind, shake or sprinkle in pepper until there is an even coat across the bowl.” This is again done in the video. The woman continues, “Next, I’m going to dip my finger into the pepper, and watch what happens.” She dips her finger in the bowl then takes it out, showing the viewer the pepper stuck to her finger. She continues, “After I wipe my finger clean on the towel, I’m going to try it again, but this time before I dip my finger back in the pepper, I’m going to put soap on it, like this.” She puts soap on her finger and says, “Once I have soap on my finger, I’m going to dip it back into the pepper, and watch what happens.” She then dips her finger back into the pepper. At this point, the pepper spreads apart from where her finger is located and when she takes her finger out of the water, there is no pepper stuck to it. Figure 1 shows a screenshot from the video of the soap-laced finger in the pepper water. The video shows this reaction three to four times from different angles. The woman wipes her finger on the towel again, and says, “Thanks for watching.” The video was about 3 min long. The video (and data associated with this study) can be viewed at https://osf.io/vrf5t/?view_only=fd96158362fe4e96b86c31d5cd1246ea.

In the Participate condition, the parent and child go through the same demonstration on the video, but are led by the experimenter using a script almost identical to what the woman in the video says: “Today you are going to do an experiment. For this experiment, you will need a bowl (a clear or light-colored bowl will work best), water, pepper, and liquid soap. You will also need a towel.” The experimenter ensured that the dyads had these materials. She then continued, “To begin, fill the bowl with water. Then grind, shake or sprinkle in pepper until there is an even coat across the bowl. Next, dip your finger into the pepper, and watch what happens. After you wipe your finger clean on the towel, try it again, but this time before you dip your finger back in the pepper, put soap on it, like this (while the experimenter mimed putting soap on her finger) and watch what happens.” Between each step, the experimenter paused to ensure that the parent and child engaged in the particular behavior.

We analyzed whether there were differences in the dependent measures described below between the children in the Watch and Participate conditions. In addition to this contrast, we also coded the ways in which parents and children interacted in the Participate condition using the same coding scheme as that of Fung and Callanan (2013); Medina and Sobel (2020). Coders watched the parent and child participate in the demonstration to determine who set the goals for the actions. Dyads were categorized as (1) *Parent Directed*, in which parents mostly set goals for engaging in and completing the demonstration. Parents in these cases usually set out all of the materials, controlled how things were manipulated, including pouring the water into the bowl, grinding the pepper in the bowl, and rubbing the soap on their children’s fingers. (2) *Child Directed*, in which parents mostly allowed children to set goals for engaging in and completing the demonstration, which involved letting the child engage in all of the steps without offering help or support, or doing so only if asked. (3) *Jointly Directed*, in which parents supported children and helped where necessary without prompting, but collaboratively engaged in goal setting and actions that moved toward completing the demonstration. The first author, blind to any other aspect of the study, and an undergraduate research assistant, blind to all hypotheses of the study, coded these data. Agreement was 93%, Kappa = 0.82, with disagreements resolved through discussion.

After the dyads watched the video or participated in the demonstration, they were given ~30s to discuss what they watched or saw. During their participation in or watching of the demonstration and throughout the 30s after, the experimenter allowed them to talk to each other about their experience. We specifically concentrated on the extent to which they generated causal utterances, measured by the percentage of the utterances generated by parents or children that were causal in nature. Two research assistants coded these utterances for causal language, as well as other linguistic utterances (see Supplementary material for the full coding scheme). Agreement was 87%, Kappa = 0.80. Disagreements were resolved through discussion with the first author.

Children were then given three other procedures during the first session: a theory of mind battery, a working memory battery, and an interview in which they were asked to reflect on their experience. These are described below.

##### Theory of mind battery

In the theory of mind battery, children were given three measures from the theory of mind scales (Wellman and Liu, 2004): Knowledge Access, Content False Belief, and Real Apparent Emotions. These were administered as described in Wellman and Liu (2004). Children were also given a measure of second-order false belief (Perner and Wimmer, 1985), using the script from that paper’s procedure section. These measures are described in detail in the Supplementary material. To score this battery, we summed the number of measures on which children responded correctly.^3^ This battery was scored by an undergraduate research assistant, blind to the hypotheses of the study. A second undergraduate assistant coded 20% of the data. Agreement was 100%.

##### Working memory battery

Children were given a series of forward and backward digit span tests. For the forward span tasks, children were told a set of numbers, and were asked to repeat those numbers back to the experimenter in the order in which they were presented. Children were first given two trials of a set of three numbers. If they responded correctly on at least one trial, they were given two trials of four numbers. If they responded correctly, the quantity was increased until children were given sets of nine. The backward span task was similar to the forward span task, except that children were instructed to list the numbers in the reverse order in which they were told. On this task, children started with a set of two numbers and proceeded up to nine numbers one at a time if they got at least one of the two trials correct. This battery was scored by an undergraduate research assistant, blind to the hypotheses of the study. A second undergraduate assistant coded 20% of the data. Agreement was 100%.

##### Reflection

Children were told that the experimenter would ask them a set of questions, and that there are no wrong answers to these questions, and that the experimenter was “just trying to learn about what you think and remember.” Children in the Participate condition were asked to tell the experimenter, “What happened in the experiment that you did with your parent?” whilst children in the Watch condition were asked to tell the experimenter, “What happened in the experiment you watched in the video with your parent?” Children were given the opportunity to respond, and the experimenter used further open-ended questions to make sure that the child talked as much as possible about their experience (e.g., “Is there anything else you want to tell me?”).

She then asked, “What did you see happen when you dipped your finger/the woman dipped her finger into the water without the soap?” and why they thought that happened, using open-ended prompts (e.g., “Do you want to tell me more?” or “Is there anything else you want to tell me?” or “I’m just trying to get all of your thoughts out of you.”). She then asked “What did you see happen when you dipped your finger/the woman dipped her finger into the water with the soap?” and why they thought that happened. Again, open-ended prompts were used to make sure that the child had every opportunity to reflect on the experience, both in terms of what was happening and why. She then asked a set of structured questions: (1) “Did what you see remind you of anything or make you think of anything?” (2) “Did you learn anything?” and (3) “Did you have fun?” If children said yes to any of these questions, she probed for the child to give them more information. Children were then prompted to tell the experimenter anything else that they saw in the experiment or video that they wanted to share. Finally, the parent was asked whether they or the child had seen the pepper demonstration previously.

Here, we focused on whether children spontaneously generated causal or relational connections in their response to the first open-ended question (“What happened in the experiment you watched/did with your parent…”) as well as whether children generated a causal explanation in terms of soap or germs in response to what happened when a finger was dipped in the pepper water without and with soap. Children received a score of 1 for each of these opportunities, for a score of 0–3. Other aspects of the coding of the reflections are described in the section Supplementary material. These reflections were scored by the second author and a research assistant, blind to the hypotheses of the experiment. Agreement was 95%, Kappa = 0.92. Disagreements were resolved through discussion.

#### Between sessions

Directly after the first session, participants were sent an Amazon gift card for $20, and a reminder for their second session. The next day, and every day until (and including the day of) their second session, the target parent was sent an email with a handwashing questionnaire. This email was automatically sent at 8 am ET. In particular, we asked parents to, “Think about the last time [their] child was in a situation where they would typically wash their hands (e.g., before eating, after using the bathroom, etc.).” Parents were then asked to choose whether the child washed, washed only with prompting, did not wash, or that they did not know. If parents indicated they washed, they were asked whether the child used soap (again clarifying if the soap use was prompted or unprompted). The full questionnaire is provided in the Supplementary material. Here, we considered two variables: the percentage of questionnaires on which parents reported that children washed their hands without prompting, and the percentage of questionnaires on which parents reported that if their children washed their hands, they used soap without being told by an adult. These two dependent variables reflect the extent to which children internalized the behavior of handwashing, and the question is whether those behaviors differed based on the participate/watch condition or among the parent–child interaction styles.

#### Second session

After a brief introduction, the experimenter prompted children to reflect on their first session experience with the activity. The experimenter then administered two additional measures: Contagion Vignettes and the Handwashing and Germ Knowledge Interview.

##### Reflection

The script for the reflection in the second session was the same as the script for the first session. We concentrated on coding the same causal utterances as in the first reflection. After completing the questions from the script used in the first reflection, the experimenter asked the child, “When do you use soap?” and children were prompted to give as many examples as they could. These reflections were coded by two research assistants, different from those who coded the first reflection. Both were blind to the hypotheses of the study. Agreement on the codes was 97%, Kappa = 0.95. Disagreements were resolved through discussion.

##### Contagion vignettes

**T**he vignettes were modeled after Blacker and LoBue (2016). Children were introduced to two characters. The experimenter shared her screen, and showed children a picture of a character with their arm in a sling or with a tissue against their red nose and a red thermometer sticking out of their mouth.

For the character with the tissue and red nose, children were told, “This is Sal. Sal has a cold, so Sal has a runny nose, a headache, and sore throat.” They were then asked three questions, (1) “How did Sal get a cold?” (2) “If Sal’s friend plays with him while he has a cold, will Sal’s friend get a cold, too?” and (3) “What if you played with Sal? Would you get a cold, too?”

For the character in the sling, children were told, “This is Danny. Danny has a broken arm, so his arm is swollen and really hurts when he tries to move it.” Again, they were asked three questions: (1) “How did Danny get a broken arm?” (2) “If Danny’s friend plays with him while Danny has a broken arm, will Danny’s friend get a broken arm, too?” and (3) “What if you played with Danny? Would you get a broken arm, too?”

Children received a score of 1 for each question they answered correctly (indicating that they gave a response that was relevant to contagion on the first question for the character with a cold and that was irrelevant to contagion on the first question for the character with a broken arm, and that both they and another person would get sick if they played with the character with a cold, but not that they would get a broken arm if they played with the friend with a broken arm). Thus, children received a score of 0–6 on this measure. These vignettes were scored by two research assistants, blind to the hypotheses of the experiment. Agreement was 89%, Kappa = 0.79. Disagreements were resolved through discussion.

##### Hand washing and germ knowledge interview

This interview consisted of a set of open-ended questions about the importance of handwashing and how germs are related to the spread of disease. Some of the questions here were modeled after those used by Conrad et al. (2020), see also Leotti et al., 2021).

1. “Why is it important to wash your hands with soap?” For this question, we first categorized whether children generated a relevant response. If they did, we coded that response as to whether it mentioned any of the following: *Behavior*, which involved keeping clean or the act of handwashing (e.g., “To keep your hands clean.”); *Self Prevention,* which involved preventing themselves from getting sick (e.g., “So I do not get sick.”); *Other Prevention*, which involved preventing illness in others (e.g., “To not spread germs to someone else.”); and *Biological Process*, which involved explicit talk about germs, germ transmission or how germs work in the body (e.g., “It gets rid of germs.”). These codes were not mutually exclusive.
2. “How do people get sick?” and (3) “What can people do to not get sick?” For both of these questions, we first categorized whether children generated a relevant response. If they did, we coded that response as to whether it mentioned any of the following: (1) Behaviors related to biological processes other than germs/contagion (e.g., not getting enough sleep, not eating healthy, etc.). (2) Behaviors related to contagion (e.g., not washing hands, getting sneezed on, etc.). (3) Physical Processes such as proximity to others (e.g., playing with someone who is sick, spreading germs to someone else, etc.) and (4) Biological Processes, such as talk about germs and how they are transmitted or work in the body (e.g., germs get into your mouth or nose, they attack your healthy cells, etc.). These codes were not mutually exclusive.
3. “Tell me everything you know about germs.” We again first categorized whether children generated a relevant response. If they did, we coded that response as to whether it mentioned any of the following: (1) A description of germs (examples include describing them as tiny, as cannot be seen, as being everywhere, as there being good and bad germs, etc.). (2) Behaviors related to germs (e.g., “We have to wear a mask to prevent them going in our mouths”). (3) Physical processes, which involved talk of physical proximity in the spread of germs (e.g., “You can spread germs through touching”) and (4) Biological processes, which includes how germs are transmitted biologically or how they work in the body (e.g., germs make us sick, they go in through our nose or mouth). Again, these codes were not mutually exclusive.
4. Coders also noted whether children ever spontaneously talked about COVID-19 or ever referred back to the pepper activity during this interview. This interview was coded by two research assistants, blind to the hypotheses of the study. Agreement was 86%, Kappa = 0.78. Disagreements were resolved by the first author.

## Results

We organize our results section around the three research questions described in the introduction. First, we consider whether there were differences between the conditions regarding how parents responded to the handwashing questionnaires between the two sessions, and among the parent–child interaction styles in the Participate condition. That is, does participating in the activity or watching the activity relate to children’s subsequent handwashing behavior, particularly regarding soap use, and are differences within the Participate condition related to the parent–child interaction style during the demonstration? Second, we consider whether the conversation that children have with their parents during and immediately following the demonstration or video as well as the reflections children have about the experience relate to their handwashing behaviors. Third, we consider whether any of these relations are mediated by children’s understanding of disease transmission, other cognitive capacities, or demographic information.

### Parent–child interaction style and handwashing behavior

There were no significant differences between the frequency of parents reporting unprompted handwashing or unprompted soap-use between the Participate and Watch overall, both |*t*(79)-values| < 0.74, both *p*-values >0.46. These was our planned comparison. All subsequent analyses should be considered exploratory.

Tables 1, 2 show the percentage of questionnaires on which parents reported that children washed their hands with or without being prompted, and the percentage of questionnaires on which parents reported that their children used soap (prompted or unprompted), looking across the three parent–child interaction styles in the Participate condition as well as the children in the Watch condition. On average, parents completed 8.23 handwashing surveys in the Watch condition (SD = 1.69, Range: 4–14) and 8.27 handwashing surveys in the Participate condition (SD = 2.20, Range 5–15). This was not a significant difference, Mann–Whitney U = 766.00, z = −0.53, *p* = 0.60.

**Table 1:** Responses to the question of whether child washed hands prompted or unprompted (standard deviations in parentheses).

		Do not know	Did not wash	Washed hands with prompting	Washed hands without prompting
Participate condition	Parent directed (*N* = 11)	7 (13)	13 (18)	35 (24)	45 (28)
	Jointly directed (*N* = 25)	1 (4)	1 (3)	39 (31)	58 (32)
	Child directed (*N* = 5)	0 (0)	5 (8)	31 (23)	63 (27)
Watch condition (*N* = 40)		3 (8)	2 (5)	36 (33)	59 (35)

**Table 2:** Responses to the question of whether used soap prompted or unprompted (standard deviations in parentheses).

		Do not know	Did not use soap	Used soap with prompting	Used soap without prompting
Participate condition	Parent directed (*N* = 11)	26 (21)	3 (11)	5 (10)	66 (32)
	Jointly directed (*N* = 25)	3 (6)	0 (0)	13 (20)	84 (22)
	Child directed (*N* = 5)	10 (6)	0 (0)	12 (13)	78 (13)
Watch condition (*N* = 40)		4 (11)	6 (16)	7 (17)	83 (26)

We first considered several aspects of the demographics of our sample. This included whether there were differences in Caregiver’s gender, age, education level, reported household income, the number of children in the home, the caregiver’s experience with science education, and their attitudes about science score. None of these demographic factors were significantly related to children’s handwashing behavior, and there were few significant relations with any of the other dependent variables of interest. Please refer to the Supplementary material for detailed analyses and the reporting of these null results.

We constructed two generalized linear models on the percentage of times parents reported their children washed their hands spontaneously and the percentage of time they used soap spontaneously, with age (in months) and parent–child interaction style across the conditions (parent-directed, jointly-directed, child-directed, and Watch condition) as the independent variables. The first model – on children’s spontaneous handwashing – revealed only a main effect of age. As children got older, their parents were more likely to report that they washed their hands spontaneously, *B* = 0.008, SE = 0.003, 95% CI [0.003, 0.013], Wald χ^2^(1) = 10.35, *p* = 0.001. The second model – on children’s spontaneous soap use when they washed their hands - did not reveal a significant effect of age, *B* = 0.002, SE = 0.002, Wald χ^2^(1) = 0.77, *p* = 0.38, but did reveal differences among the parent–child interaction styles and the Watch condition. In particular, children in the parent-directed group used soap less frequently than children in the jointly-directed group, *B* = 0.19, SE = 0.09, 95% CI [0.02, 0.36], Wald χ^2^(1) = 4.54, *p* = 0.03 and children in the Watch group, *B* = 0.17, SE = 0.08, 95% CI [0.004, 0.33], Wald χ^2^(1) = 4.03, *p* = 0.05. No other significant effects were found.

### Language and reflections

We next considered whether the explanations and causal language children heard or generated during and after they participated in or watched the demonstration influenced their handwashing behavior, as well as whether their handwashing behavior was related to the amount of causal information they generated during their reflections. Table 3 shows the percentage of causal language children heard or generated during and after they participated in the activity or viewed the video. This table also shows the causal scores on both the first and second reflection about their experience with the activity. None of variables differed across the three parent–child interaction styles and the Watch condition, all Kruskal-Wallis H(3)-values <2.69, all *p*-values >0.44 (see Supplementary material for more analyses, in particular analyses of other types of language coded during the interaction, which were all unrelated to children’s handwashing behaviors).

**Table 3:** Percentage of causal language generated by parents and children after demonstration or video and children’s causal scores on first and second reflection (standard deviations in parentheses).

		Percentage of parent causal language	Percentage of children’s causal language	Children’s causal score on first reflection (out of 3)	Children’s causal score on second reflection (out of 3)
Participate condition	Parent directed (*N* = 11)	4 (8)	8 (15)	1.45 (1.21)	0.81 (1.07)
	Jointly directed (*N* = 25)	6 (8)	10 (13)	1.48 (0.82)	1.16 (0.80)
	Child directed (*N* = 5)	4 (6)	2 (4)	1.80 (0.84)	1.20 (0.83)
Watch condition (*N* = 40)		10 (16)	9 (14)	1.48 (0.96)	1.30 (0.88)

Table 4 shows the set of zero-order correlations between the two dependent measures and these measures of language, as well as children’s age. As can be seen in the table, there was a significant correlation between the percentage of times parents reported their children washing their hands and using soap spontaneously as well as a significant correlation between handwashing and age. There was also a significant relation between the percentage of times parents reported their children washing their hands spontaneously and the amount of causal language they generated. To isolate the independent effects of these variables, we constructed a generalized linear model on the percentage of time children washed their hands spontaneously, with these three variables. Age had a unique effect on handwashing with older children reported as washing their hands spontaneously more often, *B* = 0.006, SE = 0.003, 95% CI [0.001, 0.11], Wald χ^2^(1) = 6.24, *p* = 0.01. Parents’ causal talk was also a significant predictor, *B* = 0.64, SE = 0.29, 95% CI [0.07, 1.21], Wald χ^2^(1) = 4.91, *p* = 0.03. No other variable was significant.

**Table 4:** Pearson *r*(79) values and significance levels among variables handwashing and language variables.

	Age	Unprompted handwashing	Unprompted soap usage	Parental causal language	Children’s causal language	Causal score, first reflection
Unprompted handwashing	0.34 *p* = 0.002	–				
Unprompted soap usage	0.09 *p* = 0.43	0.22 *p* = 0.04	–			
Parental causal language	0.14 *p* = 0.20	0.29 *p* = 0.008	0.10 *p* = 0.37	–		
Children’s causal language	0.21 *p* = 0.05	0.16 *p* = 0.17	−0.03 *p* = 0.81	0.46 *p* < 0.001	–	
Causal score, first reflection	0.27 *p* = 0.01	0.17 *p* = 0.13	0.29 *p* = 0.008	−0.14 *p* = 0.22	−0.03 *p* = 0.76	–
Causal score, second reflection	0.25 *p* = 0.03	0.20 *p* = 0.08	0.11 *p* = 0.31	0.01 *p* = 0.985	0.12 *p* = 0.28	0.46 *p* < 0.001

As can also be seen from Table 4, children’s unprompted soap use was correlated with their unprompted handwashing, as well their causal score on the first reflection (but not the second). To isolate the unique effects of the causal score on the first reflection and parent–child interaction style, which revealed significant differences demonstrated above, we constructed a Generalized Linear Model on unprompted soap use with these variables as independent measure. This revealed a similar pattern of results for the parent–child interaction styles, with children in the jointly-directed dyads using soap more often than children in parent-directed dyads, *B* = 0.19, SE = 0.08, 95% CI [0.02, 0.35], Wald χ^2^(1) = 4.75, *p* = 0.03 and children in the Watch condition using soap more often than those in parent-directed dyads, *B* = 0.17, SE = 0.08, 95% CI [0.01, 0.33], Wald χ^2^(1) = 4.49, *p* = 0.03. Children who generated more causal information during their first reflection also were more likely to used soap spontaneously when washing their hands, *B* = 0.08, SE = 0.03, 95% CI [0.03, 0.14], Wald χ^2^(1) = 8.26, *p* = 0.004.

## Interim summary

So far, we have found, through parent report, that the older children in our sample were more likely to wash their hands spontaneously after bathroom use or before eating. This behavior was also affected by the amount of causal language parents generated after participating in or viewing the demonstration. In contrast, there was no relation between children’s age and parents’ reports of spontaneous soap usage. Instead, soap use was related to parent–child interaction style and condition, with parent-directed children using soap less often. Moreover, the more causal information about germs or soap use that children generated during their first reflection, which did not differ among the parent–child interaction styles or conditions, related to their spontaneous soap usage. So, while older children might wash their hands more often, soap usage seems more influenced by how parents and children interact during the demonstration.

### Individual differences in handwashing behaviors

Our third question examined whether demographic factors or other individual differences were related to children’s handwashing behavior. Table 5 shows the average scores on the Digit Span Tests, Theory of Mind Battery and Contagion Vignettes. None of these measures significantly differed among the parent–child interaction styles and the Watch condition, all Kruskal-Wallis H(3)-values <2.75, all *p*-values >0.43. Children’s score on the vignettes significantly correlated with parental report about spontaneous handwashing, *r*(79) = 0.27, *p* = 0.01 as did children’s theory of mind score, *r*(79) = 0.36, *p* = 0.001 and their score on the backward digit span measure, *r*(79) = 0.35, *p* = 0.002. None of these variables significantly correlated with parental reports about spontaneous soap usage, all *r*-values <0.17, all *p*-values >0.14.

To consider the role of the vignettes and children’s theory of mind scores on parental reports of spontaneous handwashing, we constructed a new Generalized Linear Model adding these three independent variables to those that were significant in the analogous model from the previous section (age and parent causal talk). In this model, only parental causal talk was a significant predictor, *B* = 0.67, SE = 0.25, 95% CI [0.17, 1.16], Wald χ^2^(1) = 7.16, *p* = 0.007. This is consistent with the vignette score, the theory of mind score, and the score on the backward digit span all significantly positively correlating with children’s age, all *r*-values >0.50, all *p*-values <0.001.

We also considered children’s responses to the germ knowledge and handwashing questions, administered in the second session. Table 6 shows the frequency of each response type on the four questions, and the correlations between children’s responses and age. None of the response types to these questions, however, were significantly correlated to children’s handwashing behavior when controlling for age.

**Table 5:** Mean scores on theory of mind, digit span, and contagion measures (standard deviation in parentheses).

		Theory of mind score (out of possible 4)	Forward digit span score (out of possible 9)	Backwards digit span score (out of possible 9)	Contagion vignettes (out of possible 6)
Participate condition	Parent directed (*N* = 11)	1.90 (1.04)	3.95 (0.93)	3.32 (0.75)	5.00 (1.00)
	Jointly directed (*N* = 25)	2.20 (1.19)	4.36 (0.71)	3.20 (0.85)	4.56 (1.44)
	Child directed (*N* = 5)	2.00 (1.41)	4.20 (0.84)	3.30 (0.84)	5.60 (0.55)
Watch condition (*N* = 40)		2.25 (1.03)	4.40 (1.16)	3.29 (0.77)	4.83 (1.17)

**Table 6:** Number of children who generated responses of each type to questions about germ knowledge by type.

Question and type of response	Number (and percentage) of children generating this kind of response	Correlation with age
**Why do you wash your hands?**		
Behavioral: Children’s reasoning is related to handwashing and/cleaning behaviors (e.g., to keep your hands clean, when your hands are dirty, etc.)	33 (41%)	*r_s_*(79) = 0.19 *p* = 0.08
Self-preventative: Children’s reasoning is related to preventing their own sickness (e.g., so I do not get sick, so I stay healthy, etc.)	40 (49%)	*r_s_*(79) = 0.41 *p* < 0.001
Other-preventative: Preventative - Others justifications: Children’s reasoning is related to preventing sickness in others (e.g., to not spread germs to someone else, so others do not get sick, etc.)	11 (14%)	*r_s_*(79) = 0.19 *p* = 0.08
Biological process justifications: Children’s reasoning contains explicit talk of germs and how germs are transmitted and/or work within the body (e.g., it gets rid of germs, soap kills germs, etc.)	61 (75%)	*r_s_*(79) = 0.10 *p* = 0.33
**How do people get sick?**		
Behaviors related to biological processes (other than germs/contagion): Children’s response includes behaviors related to health but are not explicitly related to contagion (e.g., not getting enough sleep, not eating healthily, not going to the doctor, etc.)	15 (19%)	*r_s_*(79) = −0.04 *p* = 0.70
Behaviors related to contagion: Children’s response includes behaviors explicitly related to contagion (e.g., not washing hands, touching something dirty, getting sneezed on, etc.)	40 (49%)	*r_s_*(79) = 0.28 *p* = 0.01
Physical processes: Children’s response includes physical proximity or spreading through being near/close to someone (e.g., being near people, playing with someone who is sick, spreading germs to someone else, etc.)	34 (42%)	*r_s_*(79) = 0.36 *p* = 0.001
Biological processes: Children’s response includes explicit talk of germs and how germs are transmitted and/or work within the body (e.g., germs, bacteria, germs get into your mouth or nose, they attack your healthy cells, etc.)	28 (35%)	*r_s_*(79) = 0.18 *p* = 0.11
**What can people do to not get sick?**		
Behaviors related to other biological processes (other than germs/contagion): Children’s response includes behaviors related to preventing sickness but are not explicitly related to contagion (e.g., get enough sleep, eat healthily, go to the doctor, etc.)	27 (33%)	*r_s_*(79) = 0.05 *p* = 0.67
Behaviors related to contagion: Children’s response includes preventative behaviors explicitly related to contagion (e.g., washing your hands, sneezing into your elbow, wearing a mask, getting vaccinated, etc.)	63 (78%)	*r_s_*(79) = 0.40 *p* < 0.001
Physical processes: Children’s response includes preventing sickness through physical proximity and/or germ spreading (e.g., staying away from others when you are sick, not playing with friends, not sharing drinks, etc.)	40 (49%)	*r_s_*(79) = 0.25 *p* = 0.03
Biological processes: Children’s response includes explicit talk of germs and how germs are transmitted and/or work within the body (e.g., cleaning to kill germs and/or bacteria, washing your hands to get rid of germs, etc.)	10 (12%)	*r_s_*(79) = −0.06 *p* = 0.57
**Tell me everything you know about germs**		
Descriptors: Children’s response includes descriptions of germs (e.g., they are tiny, you cannot see them, they are everywhere, good germs/bad germs, etc.)	47 (58%)	*r_s_*(79) = 0.35 *p* = 0.001
Behaviors: Children’s responses include behaviors related to germs/germ transmission (e.g., we have to wash our hands, wearing a mask helps, etc.)	34 (42%)	*r_s_*(79) = −0.01 *p* = 0.92
Physical processes: Children’s responses include talk of physical proximity or the spread of germs (e.g., you can spread germs through touching, if you play with someone who is sick you can get sick, etc.)	23 (28%)	*r_s_*(79) = 0.25 *p* = 0.02
Biological processes: Children’s responses include explicit talk of germs and how germs are transmitted and/or work in the body (e.g., germs make us sick, they go in through our nose and mouth, etc.)	60 (74%)	*r_s_*(79) = 0.10 *p* = 0.37

Note that codes were not mutually exclusive and children could respond in multiple ways, so percentages for each question will not add up to 100.

Finally, in the Handwashing and Germ Knowledge Interview, we coded whether children ever spontaneously referred to COVID. Approximately 5% of the children did so, but there was no relation between children talking about COVID during this interview and their handwashing behavior, both |*r*(79)-values| < 0.10, both *p*-values >0.41. Further, children never referred back to the Pepper demonstration in this interview, so we did not consider this code further.

## Discussion

Getting children to learn about and engage in better hygiene behaviors is a goal for many parents and educators, particularly as it relates to recent events surrounding the COVID-19 pandemic. The current study thus examined whether exposure to a particular at-home activity that represented how using soap helps remove germs from one’s finger, affected children’s spontaneous handwashing and soap use over the following week.

Translating parent–child interaction practices from hands-on museum settings to the home, we found that how parents and children engaged in the activity together (either by participating in or watching the demonstration) had no effect on children’s subsequent spontaneous handwashing, but the way parents and children interacted during their participation in the activity related to children’s unprompted soap usage. The content of the conversation, particularly the extent to which parents used causal language during and after their viewing of the video or their participation in the demonstration, also related to whether children engaged in more unprompted handwashing behavior. Further, the amount of causal understanding children generated when they reflected on the experience immediately afterward (but not approximately a week later) related to their unprompted soap use when washing their hands. The extent to which children engaged in unprompted handwashing and soap use did not relate to children’s own knowledge of germs or disease transmission.

While we designed the study to examine differences between the participate and watch conditions, the review of the literature on digital learning might suggest that we should not have expected a general difference between these conditions. That said, of interest is the more exploratory differences among the parent–child interaction styles, with the watch condition serving as a potential baseline measure of children’s engagement in handwashing. Children whose parents set more of the goals and engaged in more directive behaviors used soap less often during their actual handwashing. These findings parallel cases in which parents taking over an interaction resulted in less engagement in that and related subsequent activities on the part of children (Medina and Sobel, 2020; Leonard et al., 2021; Sobel et al., 2021). More generally, we suspect that these parent-directed behaviors resulted in children believing they have less autonomy in the activity, which might make them less engaged in their participation.

This hypothesis is consistent with two facets of our data. First, children in the three parent–child interaction groups and the Watch condition were equivalent in age, and there was no difference among these groups on any other aspect of children’s performance (the theory of mind battery, the working memory tasks, the contagion vignettes, the causal knowledge generated in either reflection, or the percentage of causal utterances made by them or their parents after the demonstration, see Supplementary material for analyses). This suggests that no other aspect of cognition that we measured related to their soap use during handwashing. Second, because the demonstration was about the presence and absence of soap (rather than germs or handwashing) and we avoided sharing the study’s explicit purpose with parents, we would not have expected parent–child interaction scores to relate to children’s handwashing frequency, which was also evident in these data.

The other significant finding present in these data is that the more children reflected on their understanding of the causal relations inherent in the demonstration, the more likely they might have understood that the demonstration conveyed the importance of using soap during handwashing for the removal of germs from their hands. Critically, this understanding was unrelated to the parent–child interaction style in the Participate condition, and children’s understanding of disease transmission and contagion (as measured by the vignettes and the handwashing and germ knowledge interview).

This suggests the possibility that there are two independent mechanisms that relate to children’s use of soap during handwashing. The first is a more internal mechanism that relates to children’s causal knowledge of the role of using soap. Children’s own causal knowledge leads them to behave in certain ways as they explore the world (e.g., Legare et al., 2017). But of importance is that not all measures of causal knowledge related to children’s soap use; the only relation was between the amount of causal knowledge generated in the first reflection, not the measures of understanding germs or disease transmission in the second. It is possible that these latter measures did not test enough of children’s causal knowledge with sufficient sensitivity to demonstrate positive relations. More likely, however, is the possibility that understanding that germs cause certain kinds of disease transmission is not the same as inferring that the demonstration illustrated how soap use relates to removing germs from one’s hands during handwashing. This personal relation might be what is necessary for children to appreciate the importance of using soap during handwashing. Such a hypothesis is supported by Callanan et al. (2017), who found that parents who made personal connections when engaged with their children during informal learning activities had children who were more engaged by the activity. Parents’ explanatory talk, in contrast, did not relate to children’s engagement.

The second mechanism is a more external, social mechanism, which relates to how parents interact with their children during their participation in the activity. This latter mechanism potentially interacts with the former to produce the extent to which children feel they possess autonomy when engaging with the demonstration. When asked about why one should use soap or how diseases are transmitted, children access the causal knowledge inherent in the first mechanism. But when they actually engage in the real-world behavior of handwashing, the second mechanism related to their autonomy and the social interaction might be more dominant. The more that the parents do for their children during the activity, the less children feel that the activity is for them, and potentially the less they encode from it or the less they are engaged by it (see also Callanan et al., 2020, for a similar finding and similar suggestion about multiple mechanisms relating children’s causal knowledge and parent–child interaction during informal learning activities).

### Limitations and future directions

An obvious limitation of the present work is that we base our results on a small sample size, and the present investigation needs reproduction. We designed our study to contrast the Participate and Watch conditions. We did not find significant differences between conditions, but did find significant effects among the parent–child interaction styles within the Participate condition. A larger sample size is necessary to contrast the three parent–child interaction styles among one another, as well as with the Watch condition. As a result, the present results should be considered that of exploratory analyses and in need of reproduction. For instance, while we did find significant simple effects in soap use between the parent-directed and jointly-directed groups, we did not find such a difference for the child-directed group (where it would also be expected). However, because so few dyads were coded as child directed, it is critical to reproduce this study with a sample size large enough to perform more confirmatory analyses on the exploratory results reported here.

Moreover, the sample collected was predominantly White, and parents were highly educated. While none of the demographic variables that we analyzed related to our critical dependent measures, it is possible that the sample was not large enough to reveal such differences and these measures could have easily influenced the results. Reproduction of this finding with a larger sample size could also consider this limitation and explore whether there are demographic differences in the ways parents and children interact around hygiene more generally.

Finally, the main dependent variables of interest relied on parental report, which can be a problematic measure. Parents might simply respond with what they think the experimenter wants to hear, or elevate their child’s handwashing capacities. Parents might also be relying on children’s descriptions of their behavior, and children might fib about their handwashing behavior. Reproduction could also consider a laboratory-based measure, in which children are required to wash their hands, particularly to see if they spontaneously use soap.

Thus, while the arguments laid out here are grounded in both museum-based and laboratory-based investigations, they would benefit from reproduction with a larger sample using different, but related methods. Such investigations would also address another limitation of this study, which is that we relied on the natural-occurring interaction style between parents and children in the Participate condition, and did not manipulate the autonomy children might have believed they had during their participation. This could also be considered in further reproduction, much like how parent–child interaction to promote exploration or explanation can be manipulated through subtle instructions given to parents prior to their interaction with their children (e.g., Willard et al., 2019; Letourneau et al., 2021). However, one could also consider this particular limitation as a feature: Relying on the interaction style that manifested in our random sample is more representative than empirically manipulating children’s perceived autonomy. And to our knowledge, this is the first time that the coding scheme for goal-directedness, which was developed for studying museum exhibits, has been used with a remote activity in the home. We would suggest that the coding scheme transfers to this environment, which increases its application to future datasets.

## Conclusion

Previous investigations have found that formal education about soap use actively reduces disease transmission. The present study suggests that a simple, informal, at-home demonstration or video relates to children’s soap use during handwashing, at least in the short term. If parents are directive in how they set goals for their children during the activity, the children in the sample showed reduced use of soap in their own handwashing behaviors. While the effect of parent directedness might be small in this sample, it parallels numerous other findings that parent directedness reduces children’s engagement and sense of autonomy, and warrants further consideration.

In particular, an interesting caveat to this discussion is that recent findings have suggested ways of reducing parental directedness in museum settings. Sobel and Stricker (2022) showed that presenting families with prompts that encouraged more open-ended collaboration and exploration (e.g., “There is no wrong way to play.”) when they initially engaged with exhibit materials reduced parental directedness. It might be interesting to consider modifying the way in which museums present at-home activities, including their current handwashing-related activities, to increase collaborative, playful interactions and encourage causal language among parents through prompts (see also Willard et al., 2019). This could potentially contribute to the efficacy of such programming and increase both parents’ and children’s authentic engagement with a museum’s mission and content beyond the museum walls.

Finally, it might also be important to consider both whether parents’ own handwashing relates to children’s behavior, and if it changes based on their participation or viewing the activity (following Song et al., 2013). Hermida et al. (2021) demonstrated that not only did children’s beliefs about dengue fever change as a result of participating in an interaction with their parents, but parents’ beliefs changed as well. While we suspect that all the adults in our sample recognize the importance of soap use during handwashing, a visual reminder about its importance might benefit their own handwashing behavior.

## Data availability statement

The datasets presented in this study can be found in online repositories. The names of the repository/repositories and accession number(s) can be found at: https://osf.io/vrf5t/?view_only=fd96158362fe4e96b86c31d5cd1246ea.

## Ethics statement

The studies involving human participants were reviewed and approved by Brown University IRB. Written informed consent to participate in this study was provided by the participants’ legal guardian/next of kin.

## Author contributions

DS conceptualized the study, analyzed the data, and wrote the first draft. LS collected all data, designed coding schemes (with input from DS), and supervised all coding and data maintenance. All authors contributed to the article and approved the submitted version.

## Funding

This research was supported by NSF (grant numbers 1917639 and 2033368 to DS). Any opinions, findings, and conclusions or recommendations expressed in this material are those of the authors and do not necessarily reflect the views of NSF.

## Conflict of interest

The authors declare that the research was conducted in the absence of any commercial or financial relationships that could be construed as a potential conflict of interest.

## Publisher’s note

All claims expressed in this article are solely those of the authors and do not necessarily represent those of their affiliated organizations, or those of the publisher, the editors and the reviewers. Any product that may be evaluated in this article, or claim that may be made by its manufacturer, is not guaranteed or endorsed by the publisher.

## Footnotes

^1^An important point about self-determination theory (Deci and Ryan, 1985, 2000) is that it posits that such motivation stems from a set of innate psychological needs, which are not particularly based in physiological drives. While this is certainly a possible explanation, we are agnostic to this specific aspect of this account.

^2^Whether this demonstration can be used for that purpose is beyond the scope of this investigation.

^3^Because the theory of mind scale is progressive, it is also possible to score them as the lowest measure children responded to correctly (so that, for example, if children perform correctly on Knowledge Access, incorrectly on False Belief, but correctly on Real Apparent Emotions, they are not getting credit for passing the higher measure simply by chance). The significant levels of the analyses reported in the manuscript do not change if this alternative system is used.
