# Supplementary material for: Parent–child interaction during a home STEM activity and children’s handwashing behaviors
Source: Front Psychol. 2022 Nov 17;13:992710. doi: 10.3389/fpsyg.2022.992710 (PMC9712757; doi:10.3389/fpsyg.2022.992710)
Supplement: Supplementary file 1 [file Data_Sheet_1.docx]

Parent-child interaction during a home-based STEM activity relates to children’s encoding of the content in their spontaneous behavior: The case of soap-use during handwashing

Supplemental Materials

**Supplemental Methods and Coding**

**Handwashing Questionnaire**

This questionnaire was automatically sent to parents at 8:00AM ET every day in between the first and second session as a Google Form.

1) Think about the last time your child was in a situation where they would typically wash their hands (e.g., before eating, after using the bathroom, etc.) then choose an option that most accurately describes their hand washing behavior in that moment.

A) They washed their hands without a reminder from an adult

B) They washed their hands with a reminder from an adult

C) They did not wash their hands

D) I don’t know

2) If you indicated they washed their hands, please tell us if they…

A) used soap without a reminder from an adult

B) used soap with a reminder from an adult

C) did not use soap

D) I don’t know.

As reported in the main text, our dependent variables of interest were the percentage of forms parents generated in which they responded option A on each of the questions. The goal was to consider how children internalized handwashing and soap-use, which was reflected in parents reporting that they engaged in these activities on their own.

**Coding for Parent-Child Talk during and after the Demonstration (Participate Condition) or viewing the Video (Watch Condition)**

Sessions were transcribed from the Zoom video and parsed for parent and child utterances. Each utterance from both parents and children was coded into one of four types as shown in Table S1. Note that this coding scheme was only for the parent-child interaction during the participation session or after dyads watched the video, not the reflections.

Table S1. Language Codes for Parent and Child Utterances

| **Type 1: Causal Connections. Includes any of the following:** | Examples (Note, all examples taken from the transcripts) |
| --- | --- |
| **Causal Connections**: Making a statement or asking a question about how a specific action leads to a consequence. Statements should include mention of both the cause and effect of the experiment (what happened, and what made it happen) while questions should ask about the cause of a given effect, or the effect of one’s actions (and usually include “why” or “how”). | “When you put soap in, it makes the pepper spread out.”  “Why did the pepper do that when she put her finger in?” |
| **Making Predictions:** Suggesting or asking what *will happen* as a consequence of some action. | “I think it’ll work better if we put more pepper in.”  “What do you think will happen when she dips her finger in?”  *Note that this is distinct from “what if” questions that merely prompt an action (e.g., “What if we turn the pepper grinder this way?”) |
| **Connecting Personal/ Prior Experiences:** Relating the experience to some previous personal experience/memory or a piece of information with personal relevance. | “This is like the science experiment you did in school.”  “You’ve used this soap in the bathroom a million times.”  “What does this remind you of that we did last summer?”  “What else do we use pepper for?” |
| **Science Principles:** Relating the experience to a larger science principle or prior knowledge about general concepts related to science or asking information related to a science principle. | “The soap caused a reaction.”  “The soap’s viscosity is different than the water’s.”  “The pepper is kind of like germs.”  “Does the pepper sink or float?”  “What happens to the germs on your hands when you wash them?”   *Note that this can include analogies if stated generally -- “This is like what happens to the germs when you wash your hands.” |
|  |  |
| **Type 2: Talk about the Experiment. Includes any of the following:** |  |
| **Labeling or Describing, Relevant** **to Experiment:** Talking or asking about experiment materials or properties that are relevant to the steps and/or reaction. Relevant properties of the experiment include the materials used, the function of those materials, description of the steps, etc. | “She’s using pepper, water and soap.”  “Next, she’s going to put her finger in the water.”  “What do you need to wipe the pepper off your finger?”  “We added pepper to the bowl.” |
| **Labeling or Describing, Not relevant:** Naming things, asking questions, or talking about aspects of the experiment with no connection to experiment itself. | “The soap looks sticky.”  “Pepper can make you sneeze.”  “The water is so clear.”  “What color is the soap?” |
|  |  |
| **Type 3: Talk About Actions. Includes any of the following:** |  |
| **Directing Others’ Action:** Imperative statements telling the other person what to do. Statements about what someone needs to do, should do, and has to do generally fall into this category. | “Put more water in.”  “You need more pepper.”  Stop fidgeting.”  “Try it.”  “You need to wipe your finger.”  “You should try it again.” |
| **Narrating Own or Others’ Action:** Statements describing or asking what oneself or someone else did, is doing, or will do. Also includes statements phrased in the plural. | “I’m going to pour in the water.”  “I’ll do it.”  “You put in all the pepper!”  “We just put soap on our fingers.”  “Did you move it?” |
| **Open-ended question:** Asking questions that do not include a specific answer | “What do you want to do next?”  “How much more should we do?” |
| **Suggesting/Scaffolding Action:** Prompting or suggesting an action, not as an imperative statement but in a more subtle form or as a possibility rather than direct instruction. Includes asking or requesting that someone perform an action. | “Maybe there’s another way to do it.”  “What if you tried it again?”  “You can use more pepper if you need to.” “You try it.”  “Can you do it?”  “Do you want to add some more?”  “Want to try it?”  “Let’s put this over there.” |
|  |  |
| **Type 4: All other Talk. Includes any of the following:** |  |
| **Guiding Attention:** Suggesting that the other person focus on some part of the experiment through statements or questions (but without describing). | “Look and see what’s happening!”  “Watch, please.”  “Did you see what happened to the pepper?”  *Note that if they go on to describe the experiment in more detail in the same sentence, this would be coded as “Talk About Experiment” instead (e.g., “Look, the pepper moved!”). |
| **Emotion:** Expressions of emotions such as awe, frustration, pride, humor, as well as questions related to emotions. | “Wow! Cool!”  “Uh oh!”  “Did that surprise you?” |
| **Praise:** Praising the child’s actions or a child/parent asking for praise. | “Good job!”  “You’re so smart!”  “Did I do a good job?” |
| **Other:** Any utterances (statements or questions) that don’t fit into categories above (or don’t have enough information to categorize) | “Yes”  “Hmm”  “Okay”  “Maybe  “I’m hungry”  “I need to use the bathroom.”  *Note talking about being done with the experiment or moving on to the follow-up tasks should be coded as “All Other Talk” as well (e.g., “Is the video over?”, “Just wait, she’ll come back.”) |

The agreement statistics reported in the text were calculated based on whether coders agreed on the main Types of Language (Types 1-4) and not the subcodes listed in the Table (e.g., Casual Connections, Labeling or Describing, Suggesting/Scaffolding Action, Praise, etc.) The subcodes were only used internally to provide examples of the broader categories. We did not analyze these codes further, as we only wanted to examine the causal language generated by the parents and children during their participation or after they watched the video, as described in the main text. However, none of the other types of language significantly correlated with children’s handwashing behavior, all |*r*(79)-values| < .17, all *p*-values > .13.

**Theory of Mind Scales**

Three measures from the theory of mind scales were adapted from their descriptions in Wellman and Liu (2004). These are the Knowledge Access task, Contents False Belief task, and Real-Apparent Emotions task. The fourth measure given to children was the Ice Cream task, a measure of second-order false belief, described initially by Perner and Wimmer (1985).

***Materials***

For the knowledge access task, children were shown an opaque box, which contained a small rubber ducky. For the false belief measure, children were shown a Crayola crayons box, which contained candles. Both of these were held by the experimenter and shown to children via the camera. For the Real-Apparent emotion test, children saw on their screen (using the screen share function in Zoom) three schematic faces. One was smiling; one was frowning, and one had a neutral expression. They also saw a picture of a boy who was facing a set of four other children.

For the ice cream task, children saw a set of cartoon pictures in sequence. The first depicted two child characters (one boy, one girl). The second depicted those characters with an ice-cream man. The third was the same as the second, but with a speech caption for the girl that said, “I’m going home to get money!” The fourth depicts the girl walking to a house alone. The fifth depicts the ice cream man and boy, with the ice cream man talking to the boy saying, “I’m going to the school to sell ice cream!” The sixth depicts the ice cream man talking with the girl who is walking away from the house. The seventh depicts the girl and the ice cream man at a school. The eighth and final picture shows the boy at the house with a female adult character, who is talking to the boy.

***Procedures***

**Knowledge Access.** Children were shown an opaque box and asked what they thought was inside it. Children usually responded to this question by saying, “I don’t know” or by showing some kind of uncertainty. The experimenter then said, “OK, let’s open it and find out.” Having done so, the experimenter revealed that there was a small rubber duck in the box, and pointed this out to the child. The rubber duck was placed back inside the box and the box was closed. Children were then asked what was inside the box. The experimenter then said, “My friend Polly has never seen inside this box. Does Polly know what is inside the box?” After children responded to this question, they were asked, “Did Polly see inside the box?” To be counted as correct on this measure, children had to respond correctly to these last two questions.

**Contents False Belief.** Children were shown a Crayola crayon box. They were asked what they thought was inside the box. Children usually responded to this question by saying, “crayons” or some kind of synonym, however if they said something different, they were not given corrective feedback. The experimenter then opened the box and revealed the candles inside. Children were told, “Look, there are candles inside the crayon box.” After closing the box, the experimenter asked children what was really inside the box. This question was repeated until children responded that there are candles in the crayon box (with corrective feedback if children responded incorrectly). The experimenter continued, “My friend Peter has never seen inside this box. What will Peter think is in the box? Crayons or Candles?” After children responded, the experimenter asked, “And did Peter see inside the box?” To be counted as correct, children had to respond correctly to these last two questions.

**Real-Apparent Emotions.** To administer this procedure, the experimenter shared her screen, which showed three cartoon faces – one smiling, one neutral, and one frowning. The experimenter asked children to describe each of these faces, and reiterated that the smiling face was happy, the frowning face was sad, and the neutral face was “not quite happy, and not quite sad, so it’s an OK face.” The experimenter continued, “I’m going to tell you a story about a boy and I’m going to ask you how the boy really feels inside and how he looks on his face. So, the boy might really feel the same on the inside and on his face, or he might feel one way on the inside, but a different way on his face. So, I’m going to want you to tell me how he really feels inside and how he looks on his face.”

The experimenter then switched the slide to a picture of a boy facing a set of children and said, “This is a story about Matt. Matt’s friends were playing together and telling jokes. One of the older children, Rosie, told a mean joke about Matt. Everyone laughed. Everyone thought it was funny, but not Matt. But Matt didn’t want the other children to see how he felt about the joke, because they would all call him a baby. So, Matt tried to hide how he felt.” The experimenter then asked two memory questions. The first was, “What did the other children do when Rosie told a mean joke about Matt?” The second was, “In the story, what would the other children do if they knew how Matt felt?”

Then the three faces appeared on the screen next to the picture of Matt. Children were asked, “How did Matt really feel when everyone laughed? Did he feel happy, sad, or OK?” After children responded, they were asked, “How did Matt try to look on his face when everyone laughed? Did he try to look happy, sad, or OK?” To be counted as correct, children had to state that Matt felt worse than how he looked on his face.

**Ice Cream Task.** The experimenter continued sharing her screen, and put up the first picture of the ice cream task. The eight pictures used in this task are described in the Materials section above. The script for the story was as follows, “This is John and Mary. John and Mary are at the park. While they are there, the Ice Cream Man comes. And Mary wants to buy ice cream, but she doesn’t have any money. The Ice Cream Man tells Mary and John that he’s going to be at the park all day. So, Mary goes home to get her money. While Mary is away, the Ice Cream Man tells John that he’s going to go to the school to sell his ice cream because there is no one in the park to buy ice cream. So, the Ice Cream Man leaves, and John stays at the park. While the Ice Cream Man is driving to the school, he meets Mary walking back to the park and tells her that he’s going to the school to sell ice cream. So, Mary and the Ice Cream Man go to the school. A little while later, John goes to Mary’s house to find Mary. He asks her mom where she is, and her mom says that she went to buy ice cream.” Children are then asked, “Where will John look for Mary?” and to justify their response. She also asked children, “Does Mary know that the Ice Cream Man is at the school?” and “Does John know that the Ice Cream Man talked to Mary?”

To be counted as correct, children have to say that John will look for Mary at the park, because that’s where he thinks she is (or that he doesn’t know that she knows the Ice Cream Man is at the school) as well as respond correctly on the last two questions.

**Overall Scoring.** To generate a theory of mind battery score, performance on these four tasks were summed to create a score from 0-4.

**Results**

**Demographic Analyses**

The demographics of the sample are described in the main manuscript. In addition to children’s age and gender, we measured the caregiver’s age, gender, level of education, number of children in the household, and household income, as well as a science education score, which was based on the one used by Callanan et al. (2020). This score was calculated as follows. If the parent did not have a college degree, they were assigned a score of 0. If parents had a BA in a non-STEM-related field (as defined by the United States National Science Foundation), they were assigned a score of 1. If parents had a BA in a STEM-related field, they were assigned a score of 2. If parents had an advanced degree in a STEM-related field, they were assigned a score of 3. Parental responses to the Attitudes towards Science questionnaire (Szetcher & Carey, 2009) were also reported. Table S2 shows the correlations between these demographic data and scores on the two handwashing questionnaire measures. Because of the ordinal nature of many of our demographic questions, Spearman correlation statistics were used. However, no significance level reported below changes when Pearson correlation statistics are used.

Table S2: Correlations between demographic factors and handwashing questionnaire

|  | Caregiver Gender | Caregiver Age | Education Level | Household Income | # of Children | Science Ed Score | Attitudes towards Science Score |
| --- | --- | --- | --- | --- | --- | --- | --- |
| Wash Hands without Prompting | *r_s_*(79) = -.01  *p* = .91 | *r_s_*(79) = -.03  *p* = .78 | *r_s_*(79) = .21  *p* = .052 | *r_s_*(73) = .06  *p* = .62 | *r_s_*(79) = .09  *p* = .44 | *r_s_*(79) = .08  *p* = .48 | *r_s_*(79) = -.05  *p* = .65 |
| Use Soap without Prompting | *r_s_*(79) = -.05  *p* = .65 | *r_s_*(79) = -.10  *p* = .39 | *r_s_*(79) = -.11  *p* = .35 | *r_s_*(73) = .05  *p* = .70 | *r_s_*(79) = .06  *p* = .61 | *r_s_*(79) = -.01  *p* = .92 | *r_s_*(79) = -.10  *p* = .39 |

As can be seen in the table, none of the demographic variables significantly correlated with either of the questions from the handwashing questionnaires. Parental education level was marginally correlated with the frequency with which children washed their hands without prompting, which might be reflexive of socioeconomic status playing a role in children’s handwashing participation (with children from higher SES backgrounds more likely to wash their hands in observational studies, see Agustina et al., 2019). However, this is a speculative interpretation (and one that is not supported by the null result with household income).

We next considered whether there were any differences in the demographics between the *Participate* and *Watch* conditions or among the three parent-child interaction styles within the Participate condition. None of these analyses were significant, all Kruskal-Wallis H(3)-scores < 5.24, all *p*-values > .15.

Finally, we examined whether these demographic factors related to other measures coded in the study. This includes the proportion of causal language parents and children generated after they watched or participated in the activity, the amount of causal knowledge children generated in their reflections during the first or second session, their scores on the forwards and backwards digit span, and their theory mind score. The proportion of causal language parents and children generated and the causal knowledge children generated during their first reflection did not significantly correlate with any of the demographic factors we measured, all *r_s_*-values < |.19| all *p*-values > .11. Performance on the digit span measures were also not significantly correlated with any of the demographic factors, all *r_s_*-values < |.19| all *p*-values > .09.

The amount of causal knowledge generated during the second reflection was significantly, negatively correlated with parents’ responses to the Attitudes towards Science score, *r_s_*(71) = -.23, *p* = .05. This indicated that parents with more positive attitudes towards science had children who generated less causal information during their second reflection.

The theory of mind score showed two significant correlations with the demographics. Children tested with their mothers showed higher scores than children tested with their fathers, *r_s_*(79) = -.24, *p* = .03. Moreover, there was a significant positive correlation between children’s theory of mind score and parental education level, *r_s_*(79) = .25, *p* = .02. No other significant correlations were found.

It is difficult to interpret these findings, given that in some cases there is not an obvious explanation for the result. Because of this, we would suggest that all should be replicated to ensure that they are not simply due to Type I error.

**Parent-Child Interaction Styles**

Given that one of the main findings reported in the manuscript is that parent-child interaction style related to children’s soap use during handwashing, it is important to consider whether the parent-child interaction style related to any other facet of performance on these measures. Table S3 shows the statistical analysis for differences among the three parent-child interaction styles in the Participate condition and the Watch condition for the other variables of interest. The table also considers whether there are differences in the three parent-child interaction styles, or because the parent-child interaction styles were not uniformly distributed, and one cell (child-directed dyads) has many fewer cases, the analysis when that group is not included. As can be seen from the table, no significant differences were found.

**First Reflection**

Here, we report performance to the final three questions asked during the first reflection: 1) “Did what you see remind you of anything or make you think of anything?” 2) “Did you learn anything?” and 3) “Did you have fun?” Forty-six of the 81 children (57%) responded yes to the first question (SD = 49). Sixty-one children responded yes to the second question (76%^^[[1]](#footnote-1)^^) and 78 children responded yes to the third question (96%). The second and third question did not significantly correlate with responses to the handwashing questionnaire, all *r_s_*(79)-values < .19, all *p*-values > .10. Stating that the activity reminded them of something significantly correlated with the percentage of times parents reported that children washed their hands without prompting, *r_s_*(79) = .26, *p* = .02, but not that they used soap, *r_s_*(79) = .05, *p* = .63. However, this first finding was only marginally significant when controlling for age, *r_s_*(78) = .21, *p* = .06.

Table S3. Results of the (nonsignificant) Kruskal-Wallis H analyses of group differences based on parent-child interaction style.

|  | PCI Style and Watch Condition | PCI Style in Participate condition only | PCI Style and Watch condition without Child-Directed group |
| --- | --- | --- | --- |
| Age of children | *H*(3) = 2.43,  *p* = .49 | *H*(2) = 2.29,  *p* = .32 | *H*(2) = 0.22,  *p* = .90 |
| Forward Digit Span | *H*(3) = 1.68,  *p* = .64 | *H*(2) = 1.85,  *p* = .40 | *H*(2) = 1.33,  *p* = .51 |
| Backwards Digit Span | *H*(3) = 0.10,  *p* = .99 | *H*(2) = 0.07,  *p* = .97 | *H*(2) = 0.04,  *p* = .98 |
| Theory of Mind Score | *H*(3) = 0.83,  *p* = .84 | *H*(2) = 0.42,  *p* = .81 | *H*(2) = 0.65,  *p* = .72 |
| Causal Knowledge in First Reflection | *H*(3) = 0.75,  *p* = .86 | *H*(2) = 0.82,  *p* = .66 | *H*(2) = 0.33,  *p* = .85 |
| Causal Knowledge in Second Reflection | *H*(3) = 2.69,  *p* = .44 | *H*(2) = 1.63,  *p* = .44 | *H*(2) = 2.68,  *p* = .26 |
| Causal Talk of parents during/after activity | *H*(3) = 0.91,  *p* = .82 | *H*(2) = 0.85,  *p* = .66 | *H*(2) = 0.63,  *p* = .73 |
| Causal Talk of children during/after activity | *H*(3) = 1.61,  *p* = .66 | *H*(2) = 1.62,  *p* = .44 | *H*(2) = 0.23,  *p* = .89 |
| Disease Transmission Vignettes | *H*(3) = 2.75,  *p* = .43 | *H*(2) = 2.62,  *p* = .27 | *H*(2) = 0.11,  *p* = .95 |

If children said that the activity reminded them of something, we asked them what. Eleven of the 46 children who responded affirmatively (24%) stated that the activity reminded them of something relevant to handwashing. However, this factor did not significantly correlate with children’s handwashing behaviors, all *r_s_*-values < .17, all *p*-values > .14. It did, however, significantly correlate with the amount of causal knowledge children generated in both the first, *r_s_*(79) = .30, *p* = .007, and second reflection, *r_s_*(79) = .27, *p* = .01. This finding is consistent with other research that suggests children who make personal connections to their experiences encode more of the causal information inherent in those experiences (e.g., Callanan et al., 2017, 2020).

1. One child was not asked this question because of experimental error. [↑](#footnote-ref-1)
